# Complete mitochondrial genome and the phylogenetic position of the zebra shark (*Stegostoma fasciatum*)

**DOI:** 10.1080/23802359.2015.1137846

**Published:** 2016-02-03

**Authors:** Hao Chen, Xiao Chen, Weiming Ai, Junjie Wang

**Affiliations:** a Department of Marine Biotechnology, School of Life Science, Wenzhou Medical University, Wenzhu, Zhejiang, PR China;; b Key Laboratory of Tropical and Subtropical Fishery Resource Application and Cultivation, Ministry of Agriculture, Pearl River Fisheries Research Institute of Chinese Academy of Fishery Sciences, Guangzhou, Guangdong, PR China

**Keywords:** Orectolobiformes, *Stegostoma fasciatum*, Stegostomatidae

## Abstract

The complete mitochondrial genome of the zebra shark (*Stegostoma fasciatum*) was first determined in this study. The total length of this circle DNA was 16 658 bp, consisted of 37 genes with typical gene order in vertebrate mitogenome. Its nucleotide content was 34.1% A, 25.9% C, 12.5% G and 27.5% T. This mitogenome had 23 bp short intergenic spaces located in 10 gene junctions and 54 bp overlaps located in 11 gene junctions. In the protein-coding genes, two start codons (GTG and ATG) and two stop codons (TAG and TAA/T) were found. The 22 tRNA genes ranged from 66 bp (tRNA-*Cys*) to 75 bp (tRNA-*Leu1*). The phylogenetic result showed that *S. fasciatum* was clustered with the whale shark *Rhincodon typus*.

The zebra shark (*Stegostoma fasciatum*) (Stegostomatidae, Orectolobiformes) is found on sand, rubble or coral bottoms of the continental and insular shelves in the tropic, of Indo-West Pacific Oceans (Compagno 1984; Randall et al. 2003). The molecular and genetic research of this species was rare. In this study, we first determined the complete mitochondrial genome of *S. fasciatum*, and analyzed the phylogenetic relationship of the sharks in Orectolobiformes fishes.

Tissue of *S. fasciatum* (voucher no. RN2012122333) was collected from Ranong, Thailand. The experimental protocol and data analysis methods followed Chen et al. (2014). Excluding *S. fasciatum* and the outgroup *Chimaera monstrosa*, five species of Orectolobiformes, as well as each two species of Carcharhiniformes, Heterodontifomes and Lamniformes, with the complete mitogenomes available in the Genbank, were selected to construct the phylogenetic tree. The Bayesian method was fulfilled with the GTR + I+G model by three partitions of the mitogenome: 12S and 16S rRNA genes, and the first and second codons of the 12 protein-coding genes (except the light-strand encoded *ND6* gene).

The total length of the complete mitochondrial DNA of *S. fasciatum* was 16 658 bp (Genbank accession no. KU057952). Its nucleotide base composition was 34.1% A, 25.9% C, 12.5% G and 27.5% T. This circle molecular had a typical mitogenomic organization and gene order as most vertebrates, containing 13 protein-coding genes, 22 tRNA genes, two rRNA genes and one non-coding control region. Whole mitogenome had 23 bp short intergenic spaces located in 10 gene junctions and 54 bp overlaps located in 11 gene junctions. The 13 protein-coding genes used two start codons (GTG and ATG) as well as two stop codons (TAG and TAA/T), and most of them shared common initial codon ATG and terminal codon TAA/T. The *COI* gene owned a non-standard initial codon GTG, which is common in vertebrates (Slack et al. 2003). The *COII*, *ND4* and *Cytb* genes were terminated with a single T, whereas the *ND1*, *ND3* and *ND6* genes were terminated with the TAG. The 22 tRNA genes ranged from 66 bp (tRNA-*Cys*) to 75 bp (tRNA-*Leu1*), among which six tRNA genes were located in the light strand, while the remaining tRNA genes were in the heavy strand. The 12S and 16S rRNA genes were located between the tRNA-*Phe* and tRNA-*Leu1* genes, separated by the *tRNA*-*Val* gene. A 38 bp inserted sequence was identified as the origin of light-strand replication (OL) between tRNA-*Asn* and tRNA-*Cys* genes with a stem-loop structure. The control region was 1000 bp, presenting a high A + T content (69.3%).

In the Bayesian tree, all nodes were strongly supported (Figure 1). Each order was monophyletic. The Charcharhiniformes was the sister to the Lamniformes. The Orectolobiformes was clustered to the (Charcharhiniformes + Lamniformes) clade, and the Heterodontiformes was the basal clade in the tree. Within the Orectolobiformes, four available families formed a (Orectolobidae + (Hemiscylliidae + (Rincodontidae + Stegostomatidae))) relationship. *Stegostoma fasciatum* was clustered with the whale shark *Rhincodon typus*.

**Figure 1.**
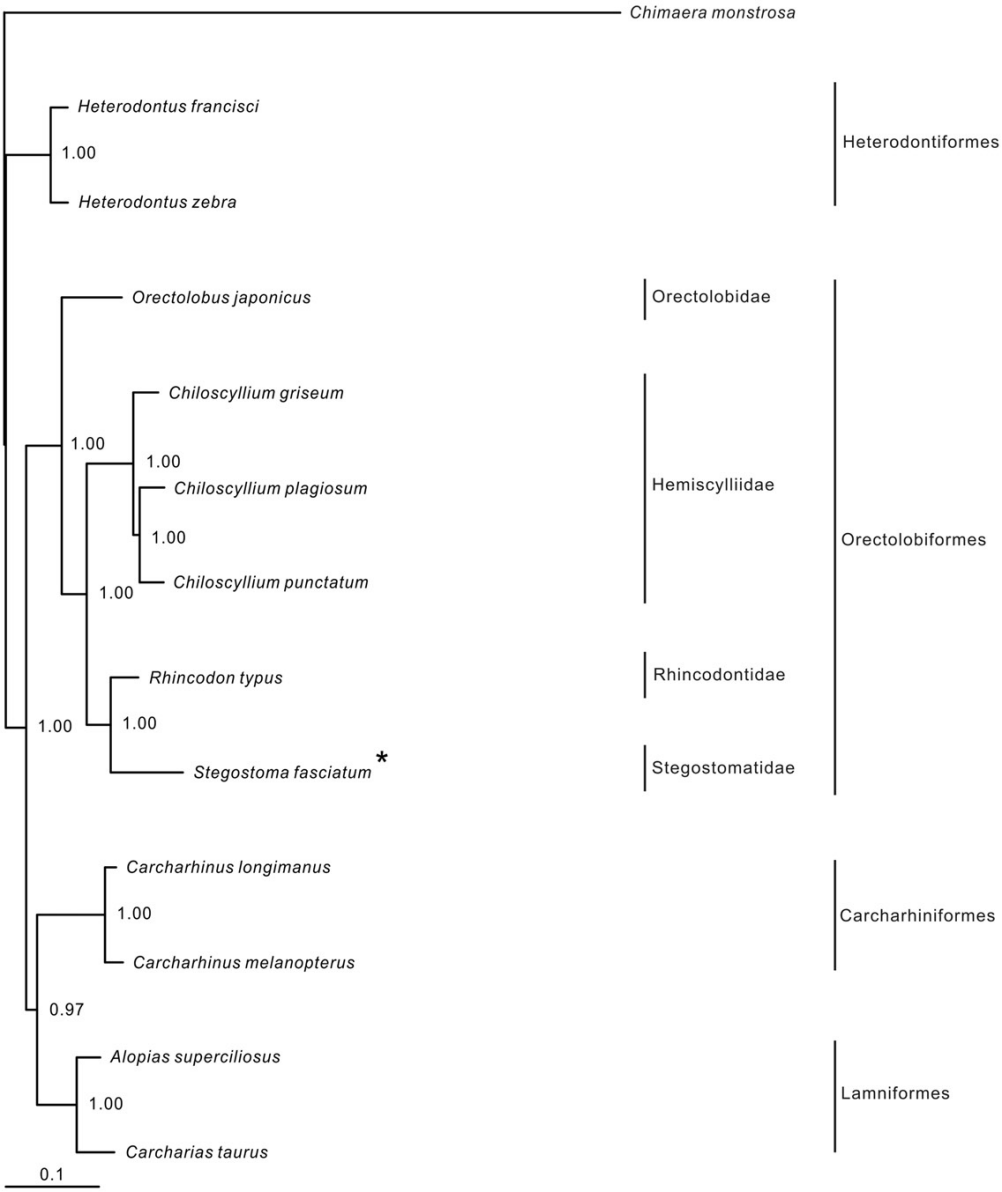
Phylogenetic position of *Stegostoma fasciatum. Chimaera monstrosa* (AJ310140.1) was selected as the outgroup. The six species from the order Orectolobiformes were *Stegostoma fasciatum* (KU057952), *Chiloscyllium griseum* (JQ434458), *C. plagiosum* (NC_012570.1), *C. punctatum* (JQ082337.1), *Orectolobus japonicus* (KF111729.1) and *Rhincodon typus* (KC633221.1). The two species from Carcharhiniformes were *Carcharhinus longimanus* (NC_025520.1) and *C. melanopterus* (NC_024284.1). The two species from Heterodontifomes were *Heterodontus francisci* (NC_003137.1) and *H. zebra* (KC845548.1). The two from Lamniformes were *Alopias superciliosus* (KC757415.1) and *Carcharias taurus* (KF569943.1).

## Declaration of interest

This study was supported by the Ministry of Science and Technology of Zhejiang Province (2013F50015), the Ministry of Science and Technology of Wenzhou City (S20140013) and Guangdong Natural Science Foundation (2015A030310002). The authors report that they have no conflicts of interest. The authors alone are responsible for the content and writing of the paper.
